# Supplementary material for: Interlaboratory Evaluation of a User-Friendly Benchtop Mass Spectrometer for Multiple-Attribute Monitoring Studies of a Monoclonal Antibody
Source: Molecules. 2023 Mar 22;28(6):2855. doi: 10.3390/molecules28062855 (PMC10053224; doi:10.3390/molecules28062855)

Supplementary Information

Interlaboratory evaluation of a user-friendly benchtop mass spectrometer for multiple-attribute monitoring studies of a monoclonal antibody

Claire I. Butré[1], Valentina d'Atri[2,3], Hélène Diemer[5,6], Olivier Colas[4], Elsa Wagner[4], Davy Guillarme[2,3], Alain Beck[4], Sarah Cianferani[5,6], Arnaud Delobel[1]

[1] Quality Assistance sa, Techno Parc de Thudinie 2, 6536 Thuin, Belgium

[2] School of Pharmaceutical Sciences, University of Geneva, CMU - Rue Michel Servet 1, 1211 Geneva 4, Switzerland

[3] Institute of Pharmaceutical Sciences of Western Switzerland, University of Geneva, CMU - Rue Michel Servet 1, 1211 Geneva 4, Switzerland

[4] Biologics CMC and Developability, IRPF, Centre d'immunologie Pierre Fabre, 5 avenue Napoleon III, 74160 Saint-Julien en Genevois, France

[5] Laboratoire de Spectrométrie de Masse BioOrganique, Université de Strasbourg, CNRS, IPHC UMR 7178, 67000 F-Strasbourg, France

[6] Infrastructure Nationale de Protéomique ProFI – FR2048, 67087 Strasbourg, France

Correspondence: alain.beck@pierre-fabre.com ; arnaud.delobel@quality-assistance.be

Table of contents

**Table S1**	List of the seven peptides that have been monitored during the MAM study.	S2
**Figure S1**	Amino acid sequence of nivolumab light chain and heavy chain.	S3
**Figure S2**	Retention times of the seven peptides monitored during the MAM study.	S4
**Figure S3**	Chromatographic separation between peptide GFYPSDIAVEWESNGQPENNYK and its deamidated variants.	S5
**Figure S4**	Determination of the deamidation of peptide GFYPSDIAVEWESNGQPENNYK in the different labs in control and high pH stressed samples.	S5

Table S1. List of the seven peptides that have been monitored during the MAM study. For each peptide, location, amino acid sequence and occurring modification (oxidation, deamidation, glycosylation, or C-terminal truncation) are reported. HC stands for heavy chain.

PEPTIDE	AMINO ACID SEQUENCE	MODIFICATION TYPE
**HC (24-38)**	ASGITFSNSGMHWVR	Oxidation
**HC (77-87)**	NTLFLQMNSLR	Oxidation
**HC (242-248)**	DTLMISR	Oxidation
**HC (282-310)**	TKPREEQFNSTYRVVSVLTVLHQDWLNGK	Glycosylation
**HC (334-353)**	GQPREPQVYTLPPSQEEMTK	Oxidation
**HC (364-385)**	GFYPSDIAVEWESNGQPENNYK	Deamidation
**HC (433-440)**	SLSLSLGK	C-term Lys truncation

Figure S1. Amino acid sequence of nivolumab A) light chain and B) heavy chain.

A) Light Chain (LC) sequence:

```
  1  EIVLTQSPAT LSLSPGERAT LSCRASQSVS SYLAWYQQKP GQAPRLLIYD
 51  ASNRATGIPA RFSGSGSGTD FTLTISSLEP EDFAVYYCQQ SSNWPRTFGQ
101  GTKVEIKRTV AAPSVFIFPP SDEQLKSGTA SVVCLLNNFY PREAKVQWKV
151  DNALQSGNSQ ESVTEQDSKD STYSLSSTLT LSKADYEKHK VYACEVTHQG
201  LSSPVTKSFN RGEC
```

B) Heavy Chain (HC) sequence:

```
  1  QVQLVESGGG VVQPGRSLRL DCKASGITFS NSGMHWVRQA PGKGLEWVAV
 51  IWYDGSKRYY ADSVKGRFTI SRDNSKNTLF LQMNSLRAED TAVYYCATND
101  DYWGQGTLVT VSSASTKGPS VFPLAPCSRS TSESTAALGC LVKDYFPEPV
151  TVSWNSGALT SGVHTFPAVL QSSGLYSLSS VVTVPSSSLG TKTYTCNVDH
201  KPSNTKVDKR VESKYGPPCP PCPAPEFLGG PSVFLFPPKP KDTLMISRTP
251  EVTCVVVDVS QEDPEVQFNW YVDGVEVHNA KTKPREEQFN STYRVVSVLT
301  VLHQDWLNGK EYKCKVSNKG LPSSIEKTIS KAKGQPREPQ VYTLPPSQEE
351  MTKNQVSLTC LVKGFYPSDI AVEWESNGQP ENNYKTTPPV LDSDGSFFLY
401  SRLTVDKSRW QEGNVFSCSV MHEALHNHYT QKSLSLSLGK
```

Figure S2. Retention times of the seven peptides monitored during the MAM study and graphical representation of the reported values. Relative standard deviation (RSD) values calculated on n = 3.

			**LAB1**		**LAB2**		**LAB3**	
			**Average**	**RSD**	**Average**	**RSD**	**Average**	**RSD**
**1**	**HC (24-38)**	Oxidation (M)	27.23	0.02	26.60	0.01	26.33	0.02
**2**		Control	31.32	0.05	30.61	0.01	30.47	0.02
**3**	**HC (242-248)**	Oxidation (M)	18.90	0.07	18.74	0.00	18.11	0.01
**4**		Control	22.13	0.06	21.80	0.01	21.31	0.01
**5**	**HC (364-385)**	Deamidation (N)	34.28	0.04	34.07	0.01	33.45	0.02
**6**		Control	34.48	0.04	34.25	0.01	33.64	0.02
**7**	**HC (334-353)**	Oxidation (M)	23.76	0.06	23.64	0.01	23.00	0.02
**8**		Control	25.99	0.04	25.83	0.01	25.30	0.02
**9**	**HC (77-87)**	Oxidation (M)	30.25	0.04	29.85	0.01	29.38	0.02
**10**		Control	35.94	0.05	35.33	0.01	35.04	0.02
**11**	**HC (433-440)**	K truncation	27.67	0.04	27.27	0.01	26.81	0.01
**12**		No K truncation	30.44	0.05	30.49	0.01	29.58	0.02
**13**	**HC (282-310)**	G2F	42.44	0.06	40.96	0.01	41.44	0.02
**14**		G1F	42.52	0.06	41.14	0.01	41.63	0.03
**15**		G0F	42.72	0.06	41.21	0.01	41.72	0.03
**16**		G0	42.87	0.05	41.35	0.01	41.88	0.03
**17**		No glycan	45.11	0.06	43.36	0.01	44.03	0.03

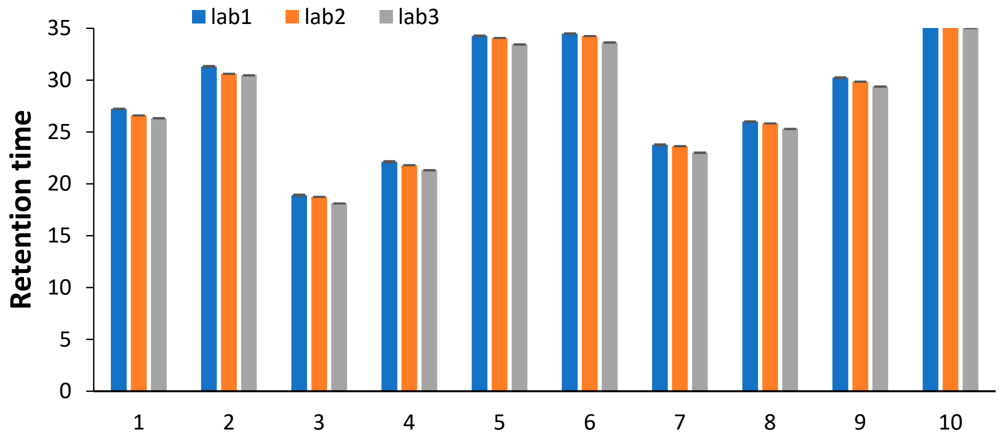

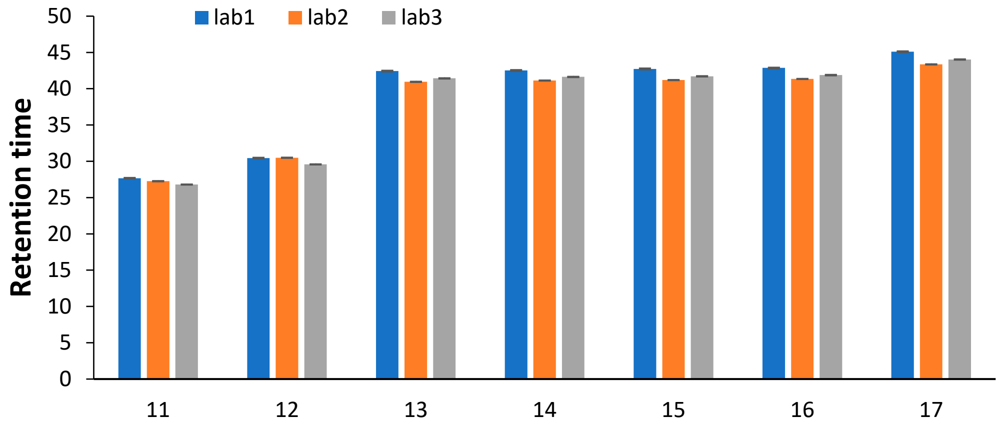

Figure S3. Chromatographic separation between peptide GFYPSDIAVEWESNGQPENNYK and its deamidated variants.

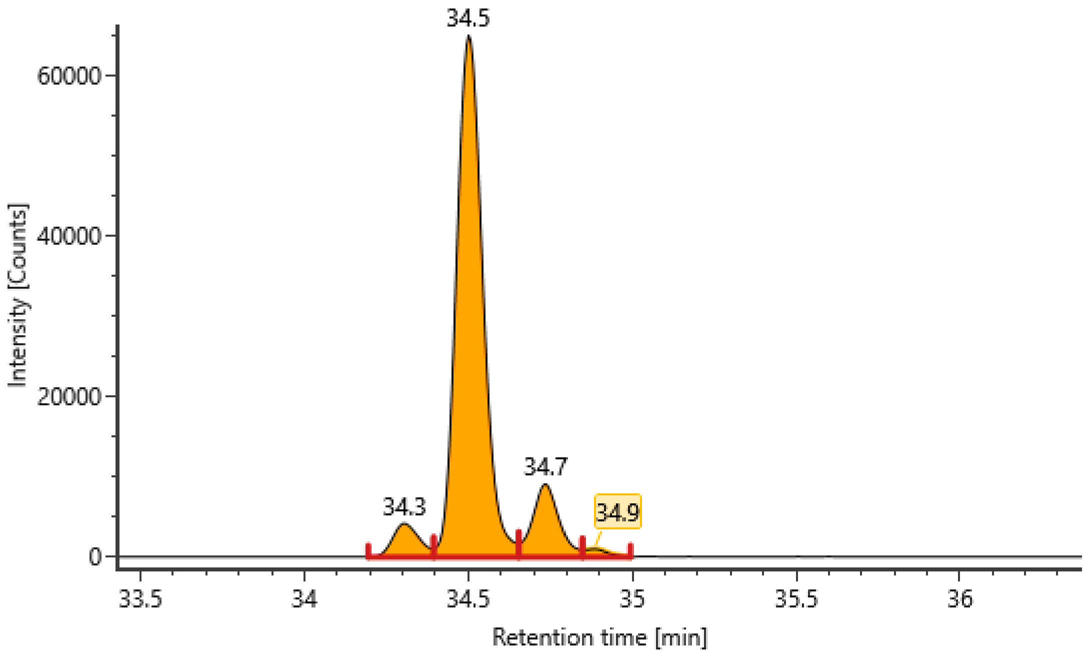

Figure S4. Determination of the deamidation of peptide GFYPSDIAVEWESNGQPENNYK in the different labs for A) control and B) high pH stressed samples.

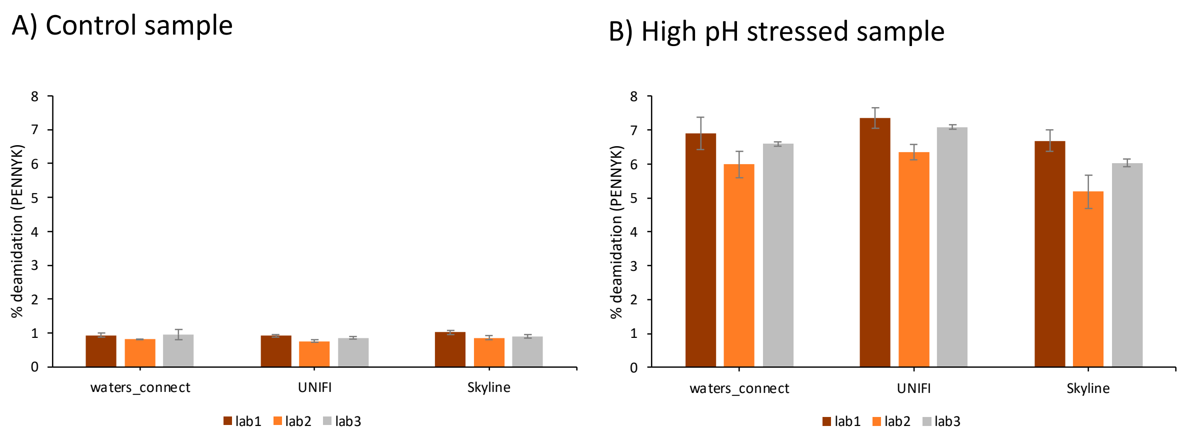

Supplement: Supplementary file 1 [file molecules-28-02855-s001.zip › molecules-2258878-supplementary.pdf]
